# Single Interdigital Transducer Approach for Gravimetrical SAW Sensor Applications in Liquid Environments

**DOI:** 10.3390/s17122931

**Published:** 2017-12-17

**Authors:** Vu Hoa Nguyen, Corinna Kaulen, Ulrich Simon, Uwe Schnakenberg

**Affiliations:** 1 Institute of Materials in Electrical Engineering 1, RWTH Aachen University, 52074 Aachen, Germany; nguyen@iwe1.rwth-aachen.de; 2 Institute of Inorganic Chemistry and JARA-Fundamentals of Future Information Technologies, RWTH Aachen University, 52074 Aachen, Germany; corinna.kaulen@ac.rwth-aachen.de (C.K.); ulrich.simon@ac.rwth-aachen.de (U.S.)

**Keywords:** SAW, impedance sensor, mass sensitive, S_11_, gold nanoparticle

## Abstract

Surface acoustic wave (SAW) devices are well known for mass-sensitive sensor applications. In biosensing applications, chemical and biochemically evoked binding processes on surfaces are detected in liquid environments using delay line or resonator sensor configurations, preferably in combination with the appropriate microfluidic devices. All configurations share the common feature of analyzing the transmission characteristic of the propagating SAW. In this paper, a novel SAW-based impedance sensor type is introduced which uses only one interdigital transducer (IDT), simultaneously as the SAW generator and the sensor element. Here, the input port reflection coefficient S_11_ is measured at the IDT instead of the commonly used S_21_ transmission forward gain parameter. Thus, a sharp and distinct peak of the S_11_ spectrum is obtained, enabling a comfortable direct readout of the sensor signal. Proof of the concept was gained by analyzing the specific binding of the 4-mercaptophenylacetic acid gold nanoparticles (MPA–AuNP) directly to the IDT surface. The corresponding binding kinetic of the MPA–AuNP on the functionalized gold surface has been analyzed and a sensitivity of 7.4 mΩ nM^−1^ has been determined.

## 1. Introduction

Surface acoustic wave (SAW) devices with interdigitated microelectrodes serving as interdigital transducers (IDTs) are commonly used in the information technology industry as electronic filters in delay line or resonator configurations [1,2]. A remarkable characteristic of SAW sensors is their sensitivity to a number of physical parameters, e.g., temperature [3,4], humidity [5,6], mass [7,8,9,10,11], and especially for the detection of (bio)chemical binding events on surfaces [8,9,10]. So far, SAW sensors are commonly used in two configurations. On one hand, in the “SAW transmission” configuration, which has become the conventional approach for mass sensitive applications, the sensor concept is based on the interaction of a propagating SAW with the adsorbed mass on a sensor surface, leading to characteristic changes of SAW velocity, resonance frequency, and amplitude [8,9,10]. Conveniently, commercially available SAW filter designs [12] can be adopted, while for gas sensing applications, SAWs in Rayleigh mode are preferred [13]. Shiokawa et al. demonstrated that Rayleigh SAWs with wave modes propagating perpendicular to the surface suffer from strong attenuation in liquid environments, which is caused by acoustic radiation losses into the fluid [14]. Thus, Y-cut lithium tantalate (LiTaO_3_) as piezoelectric substrates are successfully used for SAW sensor applications in liquid environments [15,16,17]. A waveguide layer preferably made of SiO_2_, PMMA, or polyimide, enhances the mass sensitivity, since the SAW is closely guided on the surface along the propagation path (Love wave), leading to a better interaction with the adsorbed mass [18,19,20,21]. During the last decades, this waveguide layer was optimized for applications in liquid environments [22,23]. However, in spite of the reported research progress, “SAW transmission” sensors still suffer from high signal attenuation and dispersive modes, leading to higher signal noises and a lower sensitivity. Furthermore, the additional fabrication efforts for the waveguide layer are contrary to the initial idea of the simple adoption of commercially available SAW filter designs.

On the other hand, the so-called “SAW impedance” configuration is based on the filter characteristic of SAW devices, where a change of device impedance has a direct influence on its resonance frequency. SAW impedance sensors are implemented either separately, where the SAW chip is only utilized as a simple resonator, or as connected in series with the sensitive component [24,25,26,27], forming an oscillating circuit or on-chip in a 1-port configuration. The latter is typically associated with high-Q resonators with reflectors defining a resonant cavity [28] where the resonance frequency is used as a measurement signal. Otherwise, also for sensing purposes, 1-port approaches have been designed with an SAW generating IDTs and metallic acoustic reflectors in a distinct distance as sensor elements [29,30,31,32,33], known as a 1-port SAW delay line (DL) [34]. Here, the IDT acts as an antenna and the change in the propagation characteristic of the reflected SAW is monitored. SAW impedance sensors are typically used for pressure, temperature, or viscosity measurements [35,36,37]. However, to the best of our knowledge, there are not yet any reports published on mass sensitive SAW impedance sensors in liquid environments using S_11_ as the measurement signal. 

In this paper, the proposed single IDT design is closely related to the above mentioned, 1-port SAW delay line approach, but instead of using reflectors at the end of the chip as the sensor element, the SAW-generating IDT area and the sensitive area take this function. The pivotal difference and therefore the novelty, is that the transmission characteristic is not monitored, but the input impedance of the SAW sensor is, allowing a waveguide to be neglected. Thus, a much more simplified SAW impedance sensor configuration with only one single IDT as the SAW generation, that functions simultaneously as the sensing element, is presented. Mass binding events can be monitored by continuously measuring the input impedance (S_11_ parameter). The approach is simpler in design than in the classical approaches and compared to the bulk acoustic wave devices, no separate top and backside contacts on the piezoelectric chips are needed.

At first, the sensor concept and the measurement setup are presented to point out the simplification enabled by this approach. For proof of concept, we evaluated time-resolved specific binding events of gold nanoparticles on chemically functionalized IDTs. 

## 2. Material and Methods

### 2.1. Sensor Concept

In the case of the 1-port approach, the mismatch of the input impedance can be indirectly read out using the S_11_ parameter, which represents the power reflection from the SAW sensor [24]. The S_11_ parameter can be considered as the input port reflection coefficient Γ:

(1) $$\Gamma =S_{11}=\frac{{\bar{Z}}_{in}-Z_{S}}{{\bar{Z}}_{in}+Z_{S}}$$

**Figure 1:**
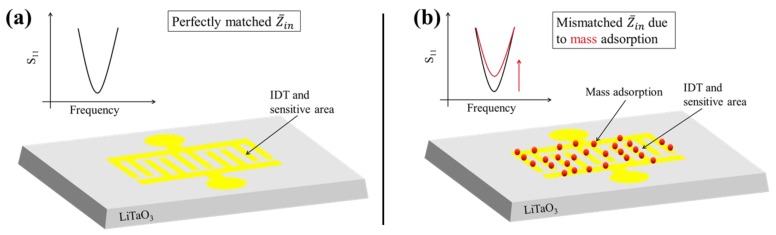
Schematic drawing of the surface acoustic wave (SAW) sensor concept. (**a**) The bare SAW sensor surface with perfectly matched ${\bar{Z}}_{in}$; (**b**) The red dots symbolize deposited mass on the interdigital transducer (IDT) surface, leading to a change of S_11_ due to the mismatch of ${\bar{Z}}_{in}$. The drawings are not to scale.

Equation (1) demonstrates the direct correlation of S_11_ to the complex input impedance ${\bar{Z}}_{in}$ and the source impedance Z_S_. This correlation has already been applied for sensor purposes, mainly for the conductivity measurements where the SAW device served as a resonant element, while the actual sensing element was part of the matching network [24] or connected in series [25,26]. However, no mass deposition application has been demonstrated yet using the S_11_ parameter as the sensor signal. Figure 1a shows the gravimetrical 1-port SAW sensor concept consisting of a piezoelectric substrate (LiTaO_3_) and one single IDT made of gold, serving simultaneously as the SAW generator and the sensitive area. In the initial situation, the input impedance ${\bar{Z}}_{in}$ is perfectly matched to the source impedance (50 Ω in our case) in order to minimize electrical power reflection. During an experiment, ${\bar{Z}}_{in}$ will change by loading mass on the IDT, leading to a mismatch condition which can be monitored by measuring the S_11_ parameter (Figure 1b).

### 2.2. Measurement Setup

**Figure 2:**
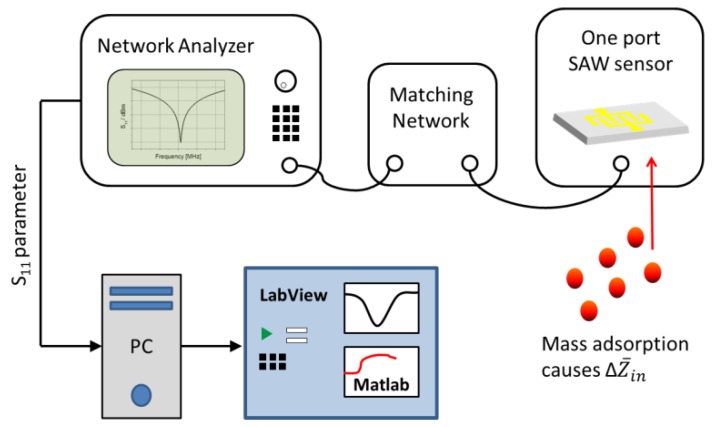
Schematic drawing of the measurement setup. A matching network matches the input impedance ${\bar{Z}}_{in}$ of the SAW device to the 50 Ω output impedance of the network analyzer. During mass deposition (red dots), an impedance mismatch Δ${\bar{Z}}_{in}$ is caused, leading to a change of the S_11_ parameter. The signal changes are monitored in real time.

The S_11_ parameter was measured by using a vector network analyzer (VNA) (HP 8712B, Agilent, Santa Clara, CA, USA), as schematically shown in Figure 2. For microfluidic applications, an electrical matching network is mandatory. A simple electrical matching network in a standard L-configuration with an inductor, capacitor, and variable capacitors was used in order to match the input impedance, prior to each experiment [38]. 

The matching network was connected upstream to the SAW device, in order to match the input impedance to the 50 Ω output impedance of the VNA, prior to each experiment. During the experiments, the S_11_ spectrum was measured from 128 MHz to 131 MHz in time intervals of 60 s. A custom-made LabVIEW program, in combination with MATLAB software, was used for automatic data acquisition and monitoring. All experiments were carried out under cleanroom conditions at a temperature of 22 °C.

### 2.3. SAW Chip Fabrication and Packaging

**Figure 3:**
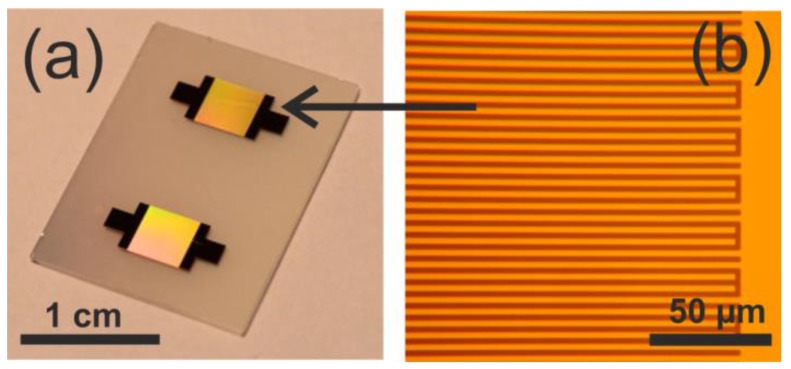
(**a**) SAW chip comprised of LiTaO_3_ substrate and two identical IDT sensor structures; (**b**) Photo of double-split fingers after lift-off process (detail). Line and space of each finger are 4 µm wide, respectively.

SAW grade 36° Y-cut LiTaO_3_ (Roditi Ltd., London, UK) was chosen as the substrate material in order to excite shear horizontal polarized SAWs with a propagation velocity of 4160 ms^−1^. Each SAW chip consisted of two identical IDT structures to carry out two independent measurements during one experiment (Figure 3a). The IDTs were prepared by sputtering 20 nm titanium as an adhesion layer and 100 nm gold (sputtering tool: Nordiko NS 2550 (Nordiko, Havant, UK), dc power of 250 W, pressure of 4.2 Pa, argon flow of 55 sccm) and were lithographically structured by a standard lift-off technique. 

A double-split finger design was chosen in order to minimize the mechanical reflection and energy loss [39]. The line and space of the double-split IDT fingers were 4 µm, respectively, to obtain a theoretical SAW resonance frequency of 130 MHz. For each IDT, 100 finger pairs were fabricated with a finger length of 3.6 mm (Figure 3b) and a total IDT width of 3.2 mm (Figure 3a).

**Figure 4:**
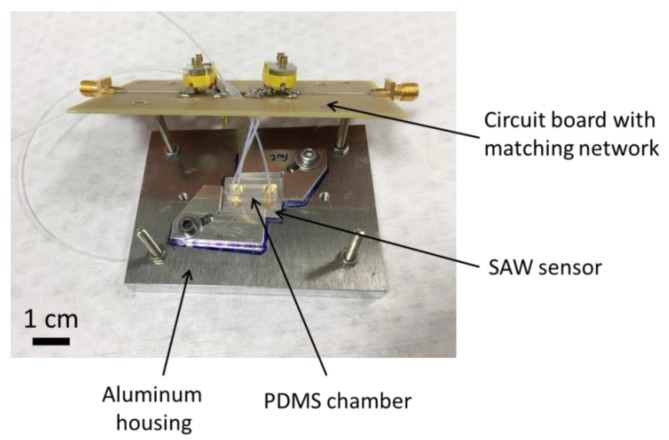
A SAW chip embedded in a custom-made aluminum fixture and covered with a poly(dimethylsiloxane) (PDMS) microfluidic chamber. The IDT was electrically connected to the matching network by spring contact probes.

The sensor chip was embedded in a microfluidic chamber, made by molding poly(dimethylsiloxane) (PDMS) with a total volume of approximately 2.8 µL for each measurement spot. The covered chip was placed in a custom-made fixture of aluminum, as shown in Figure 4. Spring contact probes were used to connect the IDT electrically. The flow rate for all fluids was adjusted to 0.2 mL h^−1^, using syringe pumps (LA-100, HLL Landgraf Laborsysteme, Langenhagen, Germany). Teflon tubes with an inner diameter of 0.3 mm were used to connect the channels and the syringe pumps. 

### 2.4. Synthesis and Characterization of 4-Mercaptophenylacetic Acid Gold Nanoparticles (MPA–AuNP)

#### 2.4.1. Materials

The following chemicals were purchased from Sigma-Aldrich Chemie GmbH, Germany, and were used as received: hydrogen tetrachloroaurate (III) trihydrate (HAuCl_4_∙3H_2_O), trisodium citrate dihydrate (Na_3_C_6_H_5_O_7_∙2H_2_O), 11-amino-1-undecanethiol hydrochloride (AUT), 4-Mercaptophenylacetic acid (MPA) and 4-(2-hydroxyethyl)-1-piperazineethanesulfonic acid (HEPES). Absolute (abs.) ethanol, hydrogen peroxide (H_2_O_2_), and ammonia solution 25% were purchased from Th. Geyer GmbH, Germany. All glassware was cleaned with aqua regia and rinsed with copious amounts of water prior to use. Ultrapure water with a conductivity <55 nScm^−1^ was used for all procedures. 

#### 2.4.2. Preparation of MPA Functionalized AuNP

Firstly, citrate stabilized AuNP were prepared according to Turkevich et al. [40]. Briefly, 50 mg of HAuCl_4_∙3H_2_O dissolved in 5 mL of H_2_O and 125 mg of Na-citrate dissolved in 12.5 mL of H_2_O were added to 250 mL of boiling water. After 20 min, the red solution was allowed to cool down to an ambient temperature. Then, 0.2 mL of a 4 mM ethanolic solution of MPA was added to 10 mL of the as-prepared citrate–AuNP solution, in order to form a self-assembled monolayer of MPA on the AuNP’s surface. After 24 h, excess ligand was removed by centrifugation at 12.000 rpm for 20 min and by redispersion in ultrapure water. After a second centrifugation step, the MPA–AuNP was redispersed in 10 mM of a HEPES buffer solution at pH 7.5. The MPA–AuNP revealed a mean particle diameter of 13.8 ± 1.4 nm, determined by Scanning Electron Microscopy (SEM). The concentration of Au was 91.92 mg L^−1^, determined by atomic absorption spectroscopy (AAS). From the AuNP, core size determined by SEM and the density of gold as the average number of gold atoms in one AuNP were calculated according to the following Equation (2).

(2) $$\frac{Au\;atoms}{AuNP}=\frac{4}{3}\pi r^{3}*\;\frac{\rho N_{A}}{M}$$

where *r* is the particle radius, ρ, the density of gold (19.32 g cm^−3^), N_A_, the Avogadro’s constant (6.022 × 10^23^ mol^−1^), and M, the molar mass of gold (196.97 g mol^−1^). By inserting the particle radius *r* = 6.8 nm in Equation (2), the average number of gold atoms in one AuNP was calculated to be 81.283. Therefore, from the Au concentration of 91.92 mg L^−1^, a molar gold concentration of 0.467 × 10^−3^ mol L^−1^ was determined, which with 81.283 Au atoms per AuNP, yielded a AuNP concentration of the Au-MPA solution of 5.7 nM.

#### 2.4.3. Functionalization of the SAW Substrates with 11-Amino-1-undecanethiol (AUT)

SAW chips were cleaned in a 1:1:6 mixture of NH_3_/H_2_O_2_/H_2_O or in oxygen plasma [p(O_2_) = 0.4 mbar, f = 40 kHz, and P = 75 W] for 4 min, immediately prior to the immersion in an ethanolic solution (c = 10^−3^ mol L^−1^) of AUT. After 24 h, the SAW chip was removed from the solution, washed with copious amounts of ethanol, and dried in an argon stream. 

## 3. Results and Discussion

### 3.1. Characterization of IDT Surface Functionalization and MPA–AuNP Interaction

**Figure 5:**
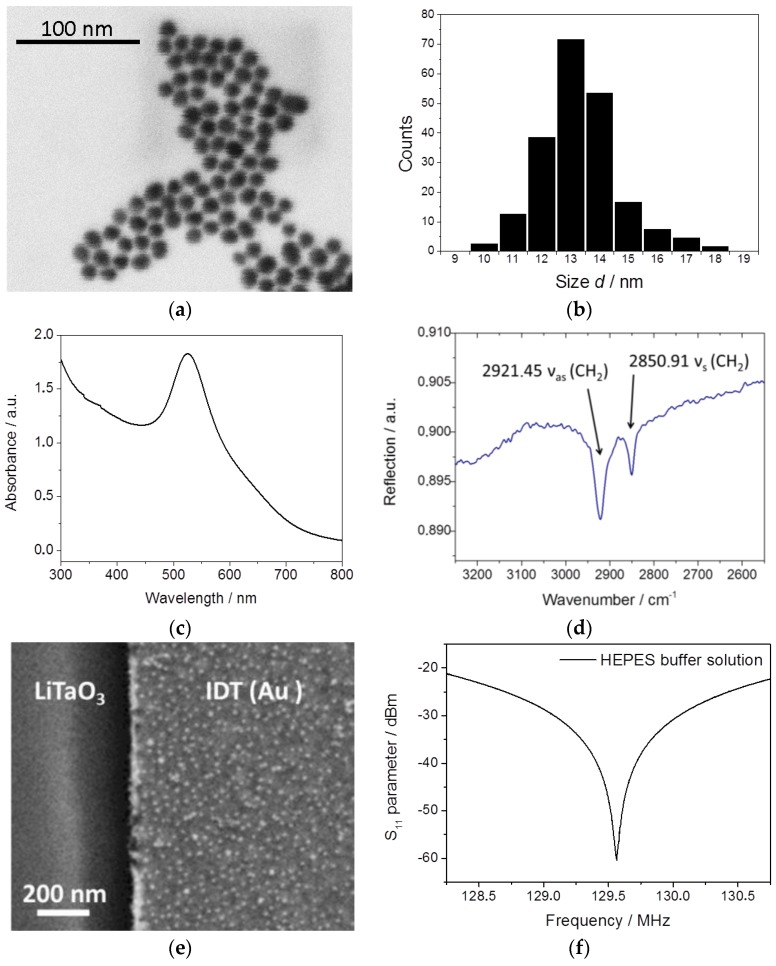
(**a**) Exemplary SEM image of 4-mercaptophenylacetic acid gold nanoparticles (MPA–AuNP); (**b**) Corresponding histogram, revealing spherical particles with a mean size of 13.8 ± 1.8 nm; (**c**) UV-vis spectrum exhibiting the typical plasmon peak at 525 nm for spherical AuNP with an average size of 15 nm; (**d**) FTIR spectrum of a self assembled monolayer (SAM) of 11-amino-1-undecanethiol (AUT) showing the absorption peaks of methyl groups indicating a successful surface modification of IDT; (**e**) SEM picture of MPA–AuNP covered IDT after applying AUT for 24 h and subsequent immersion of the SAW sensor chip in a MPA–AuNP solution for 45 min; (**f**) Measurement of a typical impedance matched S_11_ spectrum of an immersed IDT in a HEPES buffer solution.

MPA–AuNP was obtained starting from Au-citrate by performing a ligand exchange reaction in an aqueous solution. The purified particles were characterized by SEM (Figure 5a) as well as by UV–vis spectroscopy. From the SEM images, a mean particle size of 13.8 ± 1.8 nm was derived (Figure 5b). The UV–vis spectrum exhibits the typical plasmon peak at 525 nm for a spherical AuNP with an average size of 15 nm (Figure 5c). The carboxylic acid group of the MPA–AuNP is deprotonated and therefore negatively charged at pH 5 and higher, while at pH <5, protonation of the carboxylic end groups occurs, which leads to an aggregation of the particles in the acidic media, similar to mercapto-octanoic acid stabilized AuNP [41]. 

Electrostatic interaction was utilized to selectively bind the negatively charged MPA–AuNP to a positively charged gold surface. At neutral pH, the carboxylic acids are deprotonated and are thus, negatively charged, while the amine groups are protonated, and therefore positively charged. Therefore, at pH 7 ± 1, electrostatic bonding between the charged groups can be utilized. In order to facilitate this electrostatic attraction, the SAW chips were incubated in a 1 mM ethanolic solution of 11-amino-1-undecanethiol (AUT) for 24 h. After this time, a molecular monolayer of AUT had formed, which was controlled by Fourier-transform infrared spectroscopy (FTIR) (Vertex 70, Bruker, MA, USA) in reflection mode. Clear absorption bands of AUT-correlated methyl groups at wavenumbers of 2921 cm^−1^ and 2851 cm^−1^ were seen (Figure 5d) [42].

In a reference experiment, a MPA–AuNP solution (1.14 nM) in a HEPES buffer solution at pH 7.5 was drop cast on a SAW chip substrate with a preformed AUT monolayer. After 45 min incubation time at room temperature, the SAW substrates were dipped in ultrapure water to remove the MPA–AuNP solution and subsequently dried in a nitrogen stream. SEM analysis of the reference samples showed dense and homogenous adsorption of MPA–AuNP on the gold surface of the IDT structure while no AuNP were detected on LiTaO_3_ (Figure 5e). The coverage density of MPA–AuNP is 769 ± 45 particles per µm^2^, which is approximately half of the theoretically expected maximum surface coverage. In an aqueous HEPES buffer solution, an electrical double layer (EDL) surrounds the MPA–AuNP, where the negative charges of the MPA–AuNP surface are compensated by the respective counter ions. The Debye length *κ*^−1^ depicts how far these electrostatic effects persist in the medium (Equation (3)).

(3) $$\kappa^{-1}=\sqrt{\frac{\varepsilon_{r}\varepsilon_{0}k_{B}T}{2N_{A}e^{2}I}}$$

where *ε_r_* is the dielectric constant, *ε_0_* is the permittivity of free space, *k_B_* is the Boltzmann constant, *T* is the absolute temperature in Kelvins, *N_A_* is the Avogadro number, *e* is the elementary charge, and *I* the ionic strength, which is 0.01 for 10 mM of HEPES with 5 mM NaOH. The electric surface potential decays with the increasing distance from the particle surface until it is low enough to enable a close approach of another negatively charged MPA–AuNP. In previous studies, we found that negatively charged particles arrange themselves on an oppositely charged surface with a distance of 2 κ^−1^ [43]. Therefore, the theoretically derived surface coverage was obtained by dividing 90% (max. surface coverage of spheres in a densely packed monolayer) of 1 µm² by the area occupied by spheres with a r_eff_ = r_AuNP_ + 2 κ^−1^. With this approach, the theoretic maximum surface coverage of MPA–AuNP at pH 7.5 is 1864 particles per µm^2^. By this reference experiment, we showed that (i) the directed electrostatic adsorption of MPA–AuNP on AUT-functionalized gold surfaces is successful; and (ii) in order to achieve the maximum AuNP surface coverage, we need an AuNP concentration higher than 1.14 nM, or an incubation time of longer than 45 min.

### 3.2. Mass Loading and Sensor Sensitivity

The S_11_ peak amplitude of each spectrum was determined by calculating the arithmetic average value over 20 neighbor frequencies of the deepest value in order to reduce noise influence at the peak point. Figure 5f shows a typical S_11_ spectrum of an impedance matched SAW sensor during the rinsing step with the HEPES buffer solution, with the highest frequency response at approximately 129.6 MHz. It should be noted that the sharp and distinct spectrum allowed a convenient direct readout without further parameter fitting. The recorded S_11_ amplitude is used for the analyzation of the complex input impedance ${\bar{Z}}_{in}$ during mass adsorption events. In order to evaluate the sensor performance, the adsorption of MPA–AuNP was measured over time by solely switching between the nanoparticle-free HEPES buffer solution and MPA–AuNP-containing HEPES buffer solution. We observed that the influence of MPA–AuNP adsorption can be seen in the real and imaginary part of the impedance, but was best pronounced in the magnitude of the impedance. Therefore, the magnitude of the impedance was chosen to characterize the performance. 

**Figure 6:**
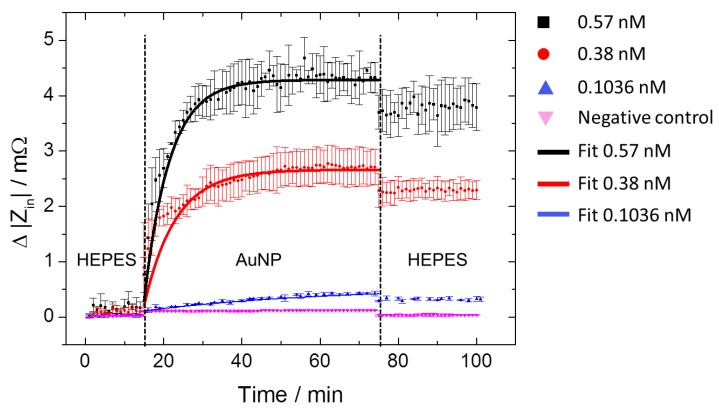
Time-resolved measurements of Δ|Z_in_| for three MPA–AuNP concentrations. The blue (upturned triangle), red (dots), and black (squares) curves correspond to the increased MPA–AuNP concentrations of 0.1036 nM, 0.38 nM and 0.57 nM, respectively. A negative control was carried out (pink downturned curve), showing no change of impedance. Exponential fit curves were calculated to determine the characteristic values (see Table 1 for fit parameters).

Figure 6 depicts time-resolved |Z_in_| changes for the three MPA–AuNP concentrations, in comparison to the negative control experiment (pink downturned triangles). In the first step, nanoparticle-free HEPES buffer solution was pumped through the microfluidic channel for 14 min to determine the initial baseline of |Z_in_|. In the next step, the HEPES buffer solution containing MPA–AuNP with concentrations of 0.1036 nM, 0.38 nM, and 0.57 nM was applied to the IDT for 60 min, followed by a final rinsing step in a nanoparticle-free HEPES buffer solution. In addition, a negative control experiment with a concentration of 0.1036 nM, but without the surface functionalization step with AUT, was carried out. For each concentration, at least three measurements were taken into account. The curves reflected a typical exponential growth kinetic where |Z_in_| increases until a saturation plateau is reached. Obviously, the plateau levels depend on the applied MPA–AuNP concentrations. In the final rinsing step, the HEPES buffer solution was applied after about 75 min to terminate the binding process and to figure out whether the selectively assembled MPA–AuNP remain adsorbed on the sensing surface. We generally observed an impedance jump when changing the media. The jump was more apparent for the switch from the MPA–AuNP solution to the final HEPES buffer treatment than for the switch from the HEPES buffer to MPA–AuNP solution. We assume that the jump solely results from the fluid change and not from the adsorption of MPA–AuNP, because the jump, especially when observed at the second media switch, occurs very fast within one measurement step. Moreover, |Z_in_| remains constant after the switch, indicating a strong electrostatic binding of the nanoparticles, without significant desorption. This assumption is supported by the negative control. In this case, only the discrete impedance jump was observed when switching between the buffer and the MPA–AuNP solution. Compared to the positive measurements with the same MPA–AuNP concentration (0.1036 nM), no increase of impedance over time (about 46 min until saturation) was monitored. Thus, the impedance jump can be considered as an offset in terms of mass adsorption analysis.

The adsorption of nanoparticles on surfaces follows first-order kinetics as the adsorption depends only on the AuNP concentration. Thus, a first-order exponential function (Equation (4)) is suitable [44,45,46] to describe the adsorption process quantitatively.

(4) $$|Z_{in}|=Z_{rel}\times \exp(-t/\tau )+Z_{Sat}$$

Here, Z_rel_ denotes the relative impedance change, Z_Sat_ denotes the input impedance change |Z_in_| at saturation, and τ denotes the exponential time constant, respectively. The values of the fitting parameters for each concentration are summarized in Table 1. 

**Figure 7:**
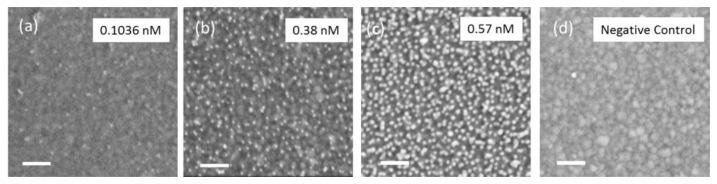
(**a**–**c**): SEM images of MPA–AuNP deposited from solutions with concentrations of 0.1036 nM, 0.38 nM and 0.57 nM, respectively, showing an increased density of the adsorbed MPA–AuNP. (**d**) A SEM image of the negative control of a SAW chip treated with 0.1036 nM MPA–AuNP but without initial AUT surface functionalization. Scale bar is 100 nm.

It turns out that the time constant τ decreases with the nanoparticle concentration which suggests that providing a higher number of MPA–AuNP clearly accelerates the saturation process. Complementary to this finding, Z_Sat_ increases with a higher nanoparticle concentration. As an obvious interpretation, we correlate the increase of Z_Sat_ to a higher MPA–AuNP density at the IDT surface, which is confirmed by a subsequent SEM analysis. SEM images of gold surfaces covered with MPA–AuNP deposited from solutions with concentrations of 0.1036 nM, 0.38 nM, and 0.57 nM reveal a particle density of 208 ± 31, 917 ± 58, and 1333 ± 106 particles per µm^2^, respectively (Figure 7a–c and Table 1). For comparison, the sensor surface of the negative control experiment shows no MPA–AuNP adsorption corresponding to the nearly constant impedance signal over time (Figure 7d). 

**Figure 8:**
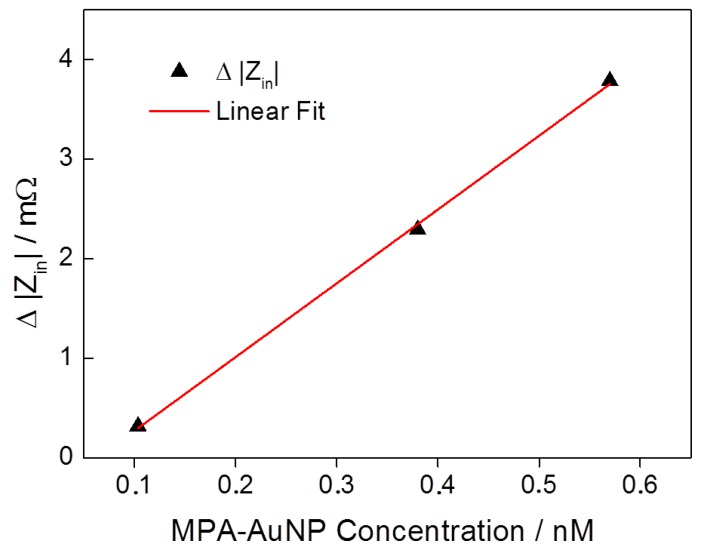
Shift of |Z_in_| with respect to different MPA–AuNP concentrations in a solution.

In order to determine the sensor sensitivity, the final change of |Z_in_| after the second HEPES buffer solution rinsing (Figure 6) has been considered and related to the results of the post SEM analyses. The results are plotted with respect to the nanoparticle concentration in Figure 8. A linear correlation between nanoparticle concentration and impedance change is emphasized by a linear fit curve (red curve). A sensitivity of 7.4 mΩ nM^−1^ has been determined. A linear relation of signal change to AuNP concentration and a comparable sensitivity has also been previously observed in a conventional delay line configuration [44]. 

The input impedance sensor signal shows a significant advantage compared to the most frequently used bench-top gravimetric biosensor approaches which monitor the dynamic characteristic of a propagating SAW [47]. As shown in Figure 5f, a spectrum with a sharp and distinct S_11_ peak is obtained whereas in delay line setups [8,9,10], the perturbing effects of the SAW propagation in spurious mode overlap or in attenuation have to be carefully considered, which hampers a simple sensor signal analysis. 

In comparison to the SAW resonator approaches which are designed to maximize the storage of energy, there is still a notable difference compared with our approach. SAW resonators benefit from a high Q-factor due to standing waves with minimal losses into the substrate or boundaries, which is achieved by a resonant cavity, defined by reflectors [13,28]. However, the latter requires very precise manufacturing in order to avoid mode jumping during operation [48,49]. In contrast, our single IDT approach is based on the electrical reflection of the single IDT and thus no energy storage is required, since the IDT is used as an antenna. Here, the electrical energy is transformed into acoustic/mechanical energy. The latter is sent out by the IDT and propagates along the substrate, while the impedance is used as the sensor signal [29,30,31,32,33,34].

The promising experimental results clearly demonstrated the proof of concept of the single IDT SAW sensor in liquid environments for gravimetrical measurements. For future applications, the influence of temperature on the sensor signal without mass loading must be characterized. Furthermore, when mass adsorption processes are analyzed, the overlaying influence of environmental parameters must be excluded. Here, the chip must be redesigned to enable differential measurements. The current chip design already comprises two single independent IDTs which can be used for differential measurements. In this context, it has to be noted that no influence of the second IDT on the measurements was observed. Measurement with diced chips (one IDT on the chip) showed no deviations in the S_11_ spectra. As expected, no SAW crosstalk was observed, since the SAW energy was absorbed in the bulk of the chip and not confined at the surface for SAW transmission by a waveguide layer. 

## 4. Conclusions

We proposed a simplified type of a SAW impedance sensor for gravimetric measurements in liquid environments. The sensor is based on a single IDT configuration to measure the input impedance mismatch during mass deposition. Compared to the transmission mode SAW sensors, the simplified design allows the sensor chip area and number of fabrication steps to be significantly reduced. Using the S_11_ parameter as the signal leads to a S_11_ spectrum with a sharp and distinct peak, allowing a direct readout of the measurement signal with no need for post data processing or complex signal amplifying electronics. The sensor performance has been quantitatively determined by measuring the specific adsorption of MPA–AuNP, revealing a sensitivity of 7.4 mΩ nM^−1^. Furthermore, kinetic analysis has been carried out, showing a lower time constant τ for higher AuNP concentrations. Although the introduced setup has not yet been optimized towards a highly sensitive gravimetric sensor, the proof of concept clearly demonstrates the feasibility of our approach of a gravimetrical sensor in liquid environments. The concept can also be deployed for chemical, biochemical, and gas sensing applications. 

With regard to commercialization aspects, the single IDT approach significantly reduces (a) the sensor chip size because a receiver IDT, as well as the waveguide layer to generate Love waves, is not needed anymore; and (b) the number of fabrication steps.

To enable future point-of-use applications, continued work should focus on the development of a handheld device where the expensive vector network analyzer (VNA) is replaced by the handheld device to measure the impedance spectrum of the SAW sensor by RF gain-phase meters [50,51]. The high frequency approach of the VNA was transferred into a quasi-static one, using simple lumped electronics without the need for impedance matching units. The single IDT approach in combination with a suitable handheld device also offers the advantage of wireless and passive measurements. 

## Figures and Tables

**Table 1:** Fit parameters determined by the first-order exponential fit function of MPA–AuNP binding for different concentrations. In addition, the final MPA–AuNP densities per µm^−2^ determined by SEM analysis are given.

Concentration (nM)	τ (min)	Z_Sat_ (mΩ)	AuNP Density (µm^−2^)
0.1036	46.2	0.32	208 ± 31
0.38	8.01	2.29	917 ± 58
0.57	6.45	3.79	1333 ± 106
